# Comprehensive Analysis of Differentially Expressed miRNAs and mRNAs Reveals That miR-181a-5p Plays a Key Role in Diabetic Dermal Fibroblasts

**DOI:** 10.1155/2020/4581954

**Published:** 2020-10-08

**Authors:** Peng Liu, Yi Zhu, Qin Li, Biao Cheng

**Affiliations:** ^1^Department of Burn & Plastic Surgery, General Hospital of Southern Theatre Command of PLA, 111 Guangzhou Liuhua Road, Guangzhou 510010, China; ^2^Huabo Post-Doctoral Research Center, Biological Pharmaceutical Research Institute, 111 Guangzhou Liuhua Road, Guangzhou 510010, China; ^3^Department of Anesthesiology, General Hospital of Southern Theatre Command of PLA, 111 Guangzhou Liuhua Road, Guangzhou 510010, China

## Abstract

A diabetic nonhealing wound causes heavy economic burden and compromised quality of life in patients. The human dermal fibroblast (HDF), which is an important kind of effector cell in the wound healing process, represents different biological behaviors in the normal and diabetic skins. Given this, we attempt to explore functional changes in diabetic skin-derived HDFs and try to find out the “hub” genes that modulate diabetic HDFs and may be the potential therapeutic targets of diabetic wound healing. We searched the GEO database for related miRNA (GSE68185, GSE84971) and mRNA (GSE49566, GSE78891) profiles. After eliminating batch effects and identifying differentially expressed genes (DEGs), we applied enrichment analyses and found that 3 miRNAs and 30 mRNAs were differentially expressed in diabetic HDFs. Enrichment analyses showed that these genes are closely related to wound healing, for example, extracellular matrix (ECM) organization, angiogenesis, cell proliferation, and migration. Subsequently, we constructed the gene correlation network of DEGs to identify hub genes by merging the protein-protein interaction network, weighted gene coexpression network, and predicted miRNA-mRNA regulatory network. Based on the gene correlation network, we identified the top 3 hub genes: miR-181a-5p, POSTN, and CDH11. Among these, POSTN is a predicted target of miR-181a-5p and is supposed to work together with CDH11 as a functional group. Finally, we verified the expression pattern of the hub genes by in vitro quantification experiments in glucose-cultured HDFs. Our study suggested that miR-181a-5p possibly plays a key role in modulation of HDF behaviors during the diabetic state. However, the effects and mechanisms of miR-181a-5p in high glucose-cultured HDFs remain to be explored in the future.

## 1. Introduction

Diabetes is a chronic metabolic disorder that brings thorough pathophysiological changes to patients, such as the high blood glucose level, disturbed water and electrolyte balance, and compromised inflammatory and immune responses. Uncontrolled diabetes can cause various changes to the skin, such as the sustained inflammatory response and ischemic state, which lead to nonhealing wound. A diabetic nonhealing wound is a common complication in diabetic patients. In severe cases, diabetic patients with nonhealing wounds in lower limbs need amputation, which account for approximately 70% nontraumatic amputations according to previous publications [1, 2].

Changes caused by diabetes occur in phagocytes, platelets, endothelial cells, keratinocytes, and importantly dermal fibroblasts. Dermal fibroblasts are the major cells which are responsible for generating connective tissue and recovering from injury. During wound healing process, fibroblasts migrate into the wound bed and produce the growth factors and extracellular matrix (ECM) to promote granulation tissue maturation. After stimulation of TGF-*β*1 secreted by macrophages, fibroblasts undergo phenotypic change, transforming to myofibroblasts which are responsible for wound contraction.

In the diabetic skin, the number of dermal fibroblasts is reduced, and the ability to secrete ECM is also compromised [3]. The reasons for impaired dermal fibroblast function in the diabetic skin wound can be explained by the unbalanced wound microenvironment, which is constituted by macrophages, endothelia, and ECM. Nevertheless, after separation from the skin, the fibroblast gene expression differences caused by diabetes persist in high-glucose in vitro conditions [4]. Therefore, the reasons for cytological changes of the dermal fibroblast should not be completely attributed to the unbalanced wound microenvironment; they also possibly underlie the abnormal genetic expression patterns of the fibroblast itself.

Several genes were found to be dysregulated in diabetic dermal fibroblasts [5]. However, among the differentially expressed genes (DEGs), which contribute most to facilitate the cytological changes of diabetic dermal fibroblasts, what are the relationships between each DEG and whether “hub” genes exist to play a pivotal role in diabetic dermal fibroblasts still remain to be explored.

In the present study, we analyzed published miRNA and mRNA expression profiling data of cultured human dermal fibroblasts (HDFs) isolated from diabetic patients. After eliminating batch effects from different studies and identifying DEGs, we applied enrichment analysis and GSEA to understand the functional changes brought by the diabetic state. Then, in the attempt to find out the “hub” genes which may master the molecular regulation of diabetic HDFs and be the potential therapeutic targets of diabetic wound healing, we constructed the gene correlation network of DEGs by merging the protein-protein interaction (PPI) network, weighted gene coexpression network (WGCNA), and predicted miRNA-mRNA target regulatory network. Finally, we tested our analysis results by determining the expression pattern of differentially expressed miRNAs and hub protein-coding genes in high glucose-cultured HDFs.

## 2. Materials and Methods

### 2.1. Data Source

We searched GEO datasets (https://www.ncbi.nlm.nih.gov/geo/) using “diabetic” and “wound OR skin OR dermal OR cutaneous” as search terms; the summary and study design of each searching result were manually reviewed. Studies comparing mRNA or miRNA expression profiles of dermal fibroblasts from diabetic patients to nondiabetic patients were included in our analysis. After study inclusion, we obtained 2 mRNA expression profiling datasets, GSE49566 (GPL8300 platform) and GSE78891 (GPL15207 platform), and 2 miRNA expression profiling datasets, GSE68185 (GPL17537 platform) and GSE84971 (GPL17537 platform). To avoid influences of confounding factors accompanied by diabetic ulcer, such as infection and tissue exposure, only fibroblast samples derived from the diabetic skin instead of diabetic ulcers were used in our analysis. Thus, a total of 11 diabetic skin-derived fibroblast (DSF) samples and 9 nondiabetic skin-derived fibroblast (NDSF) samples were included in mRNA expression profiling analysis; 7 DSF samples and 7 NDSF samples were included in miRNA expression profiling analysis. Datasets were downloaded from the GEO database; the series matrix files were used for analysis. All expression signal values of each dataset were log2-transformed before normalization.

### 2.2. DEG Identification and Functional Enrichment Analysis

To eliminate batch effects from different studies, mRNA and miRNA data were, respectively, combined using the R package of “sva” before identification of differential expression analysis. The “limma” package was used to identify DEGs. For DEG filtering, expression value fold changes (FC) with ∣log2 FC | >1 and *p* < 0.05 were used as cutoff values.

Gene ontology (GO) and Kyoto Encyclopedia of Genes and Genomes (KEGG) pathway enrichment analyses of differentially expressed mRNAs were carried out utilizing the “clusterProfiler” package. In addition, the REACTOME pathway database was also used to annotate the identified DEGs. GO and pathway terms with *p* < 0.05 were considered significantly enriched. Functional enrichment analyses of differentially expressed miRNA targets were also carried out to understand the regulatory functions of these miRNAs. Target genes of differentially expressed miRNAs were predicted by starBase v3.0 [6] (http://starbase.sysu.edu.cn/index.phps), which provides predicting results from multiple target-predicting databases. To get a generalized understanding of the DEG functions, GO terms with a level < 5 were used in analysis of miRNA targets. Venn graphs for GO terms and KEGG pathways of the miRNA targets were drawn to find the common function of the differentially expressed miRNAs.

Gene set enrichment analysis (GSEA) was applied using mRNA expression profiling data to verify major functional changes of dermal fibroblasts induced by diabetic status. The GSEA was based on the “c5.bp,” “c5.cc,” “c5.mf,” “c2.cp.kegg,” and “c2.cp.reactome” gene set collections (MSigDB, Broad Institute, Cambridge, MA, USA). The normalized enrichment score (NES) was calculated for each gene set. The permutation number of GSEA software (https://software.broadinstitute.org/gsea/index.jsp) was set to 1000, and the permutation type was set as “gene set.” Gene sets with a false discovery rate < 0.05 were considered significantly enriched.

### 2.3. Gene Correlation Network Analysis

DEGs from mRNA profiling studies were analyzed to construct the protein-protein interaction (PPI) network by using the search tool provided by the STRING database (https://string-db.org/). Interactions with a confidence score > 0.4 were included for subsequent network analysis. WGCNA analysis of differentially expressed mRNAs was carried out using the “WGCNA” package. For WGCNA coexpression network construction, the empirical power value was used. The Cytoscape network export threshold was set to 0.3. Among the DEGs, target genes of differentially expressed miRNAs were identified to constitute the miRNA-mRNA regulatory network. The miRNA-mRNA regulatory network, PPI network, and WGCNA coexpression network of DEGs were then integrated as the gene correlation network. The gene correlation network was visualized using Cytoscape software (v3.7.1; http://cytoscape.org/). The hub genes were identified by the CytoHubba plugin of Cytoscape. The CytoHubba scoring rank of each node in the gene correlation network was calculated by 12 different built-in methods, and the average rank was used to identify the hub genes. The MCODE plugin was used to extract the gene cluster which was the most correlated among the gene correlation network. To further identify the miRNAs that may control the expressions of hub protein-coding genes [7, 8], we used 2 more predicting tools of DIANA-microT-CDS [9] and miRWalk [10], other than starBase v3.0. The top 100 predicted miRNAs by each predicting tool that probably target each hub protein-coding gene were then pooled and presented as a miRNA-mRNA network by Cytoscape.

### 2.4. Cell Culture

The adult human dermal fibroblasts (HDF-a, ScienCell, USA) were obtained from ScienCell Research Laboratories. The cells cultured in Dulbecco's Modified Eagle's Medium (DMEM, Gibco, USA) were supplemented with 10% fetal bovine serum (FBS, Gibco, USA) and 1% penicillin-streptomycin solution (Gibco, UK). All cultures were maintained in a humidified incubator at 37°C and 5% CO_2_ atmosphere. The 70% confluent fibroblast cultures were maintained 24 hours in the growth medium for synchronization of the cell cycle. For further experiments, cells were treated with 3 different concentrations of glucose: 5.5 mM, 25 mM, and 60 mM for 72 hours.

### 2.5. RNA Extraction and Quantitative Real-Time Polymerase Chain Reaction (qRT-PCR) Analysis

Total RNA was extracted using the Eastep™ Super Total RNA Extraction Kit (Promega, Shanghai, China) in accordance with the manufacturer's instructions. The concentration and integrity of extracted RNA was examined by a UV spectrophotometer. Reverse transcription was performed using the RevertAid First Strand cDNA Synthesis Kit (Thermo Fisher Scientific, USA). The quantification of RT-PCR was performed with the ABI StepOne qPCR system (Life Technologies, USA) using Maxima SYBR® Green/ROX qPCR Master Mix (Thermo Fisher Scientific, USA). Primers for quantitative PCR were designed using Primer Premier 5.0 software according to the sequence data in the GenBank. The primer sequences of miR-181a-5p, miR-143-3p, miR-21-5p, CDH11, and POSTN are listed in Supplementary Materials (available here). The results were analyzed using the *ΔΔ*C[t] method.

### 2.6. Statistics

All experimental values were expressed as the mean ± standard error of the mean (SEM). The significance of differences between groups was determined using one-way analysis of variance followed by post hoc analyses. Statistical significance was considered at *p* < 0.05. Statistical analysis was performed using the Statistical Product and Service Solutions software V22.0 (SPSS, USA).

## 3. Results

The differentially expressed mRNAs were associated with extracellular matrix organization, hypoxia response, cell adhesion, proliferation, differentiation, and development.

**Figure 1:**
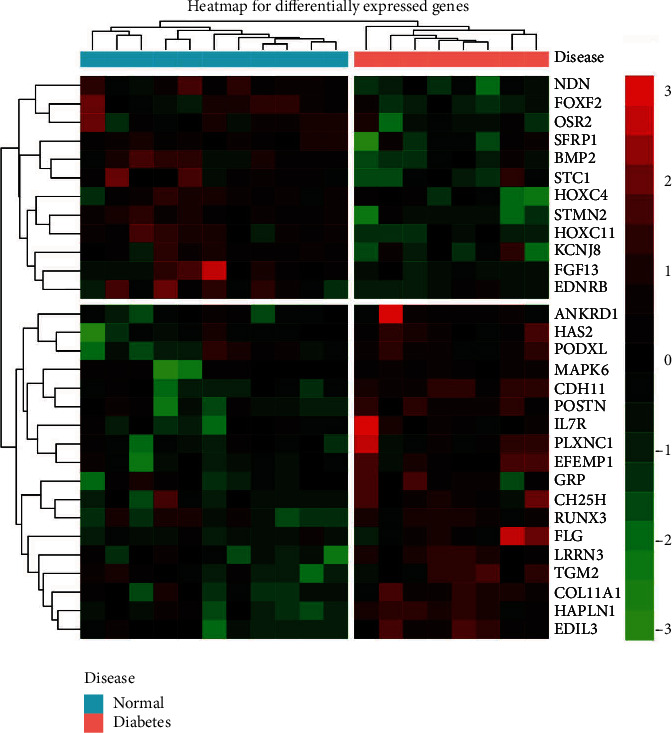
The heatmap of differentially expressed mRNAs in human dermal fibroblasts from the diabetic and normal skins. The samples used for mRNA expression profiling analysis were derived from 11 diabetic skins and 9 normal skins. A total of 30 mRNAs were significantly deregulated, with 12 mRNAs downregulated and 18 mRNAs upregulated. Red signals and green signals represent upregulated expression and downregulated expression, respectively.

**Figure 2:**
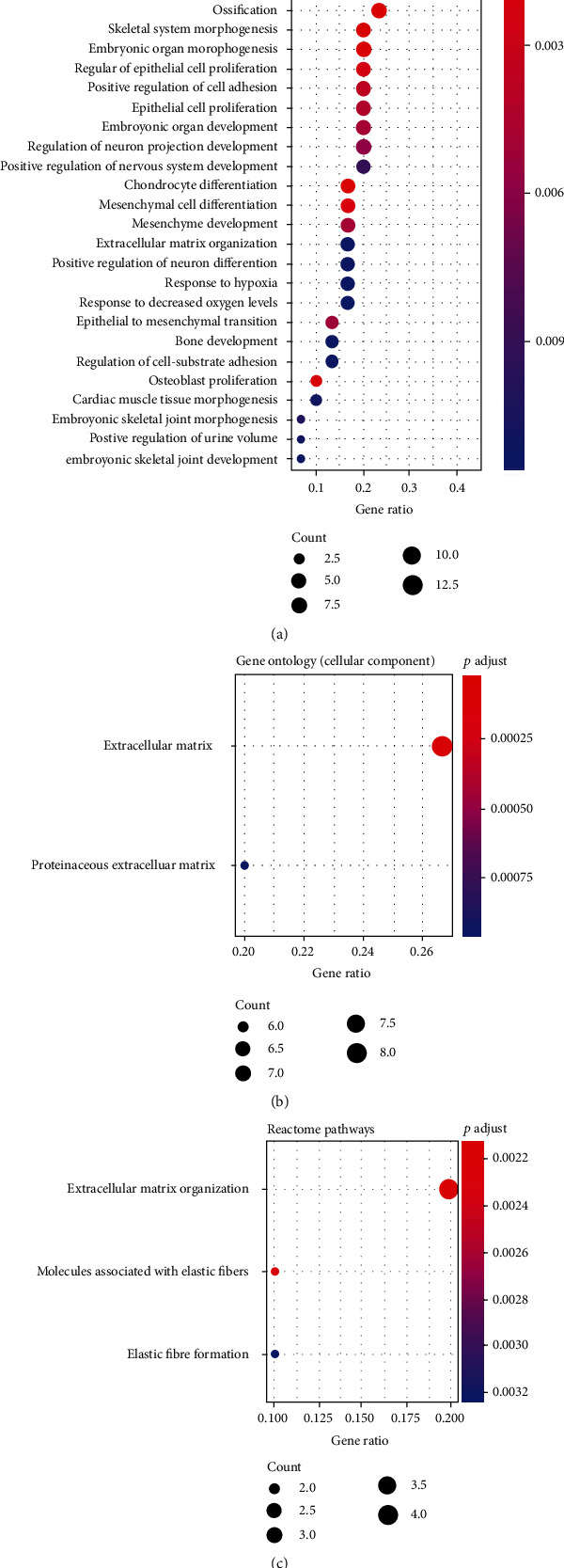
Significantly enriched GO terms and pathway terms. (a) Top 30 significantly enriched GO terms related to the biological process. (b) 2 GO terms related to the cellular component were significantly enriched. (c) 3 REACTOME pathways were significantly enriched. No GO terms related to the molecular function or KEGG pathway were significantly enriched. The gene ratio is the proportions of differentially expressed mRNAs that were correlated with a certain term. The count of mRNAs related to each term was shown by the dot size. The significance was shown by the dot color. Red indicates greater significance; blue indicates less significance.

After eliminating the batch effects of the GSE49566 and GSE78891 datasets, we combined the 2 datasets and obtained 30 differentially expressed mRNAs in cultured HDFs of diabetic patients compared with normal individuals. The heatmap of DEGs is shown in Figure 1. Among the 30 differentially expressed mRNAs, 12 mRNAs were downregulated and 18 mRNAs were upregulated. The GO terms related to the cellular component were enriched for the extracellular matrix and proteinaceous extracellular matrix. GO terms related to the biological process were mainly enriched for hypoxia response, cell adhesion, cell proliferation, differentiation, and development of multiple organ systems and tissues, for example, the skeletal system, connective tissue, and urogenital system. No GO terms related to the molecular function or KEGG pathway were significantly enriched. However, according to the REACTOME database, pathways of extracellular matrix organization, molecules associated with elastic fibers, and elastic fiber formation were significantly enriched (Figure 2).

### 3.1. Endomembrane System Activity, Ubiquitylation, and Cell Adhesion Were Common Targets of the Differentially Expressed miRNAs

Based on the analysis of the GSE68185 and GSE84971 datasets, 3 miRNAs, miR-181a-5p, miR-143-3p, and miR-21-5p, were found to be differentially expressed. All the 3 miRNAs were upregulated in diabetic HDFs. The miRNA target gene prediction utilizing the starBase V3.0 database showed 6003 target genes for miR-181a-5p, 4068 target genes for miR-143-3p, and 2781 target genes for miR-21-5p.

**Figure 3:**
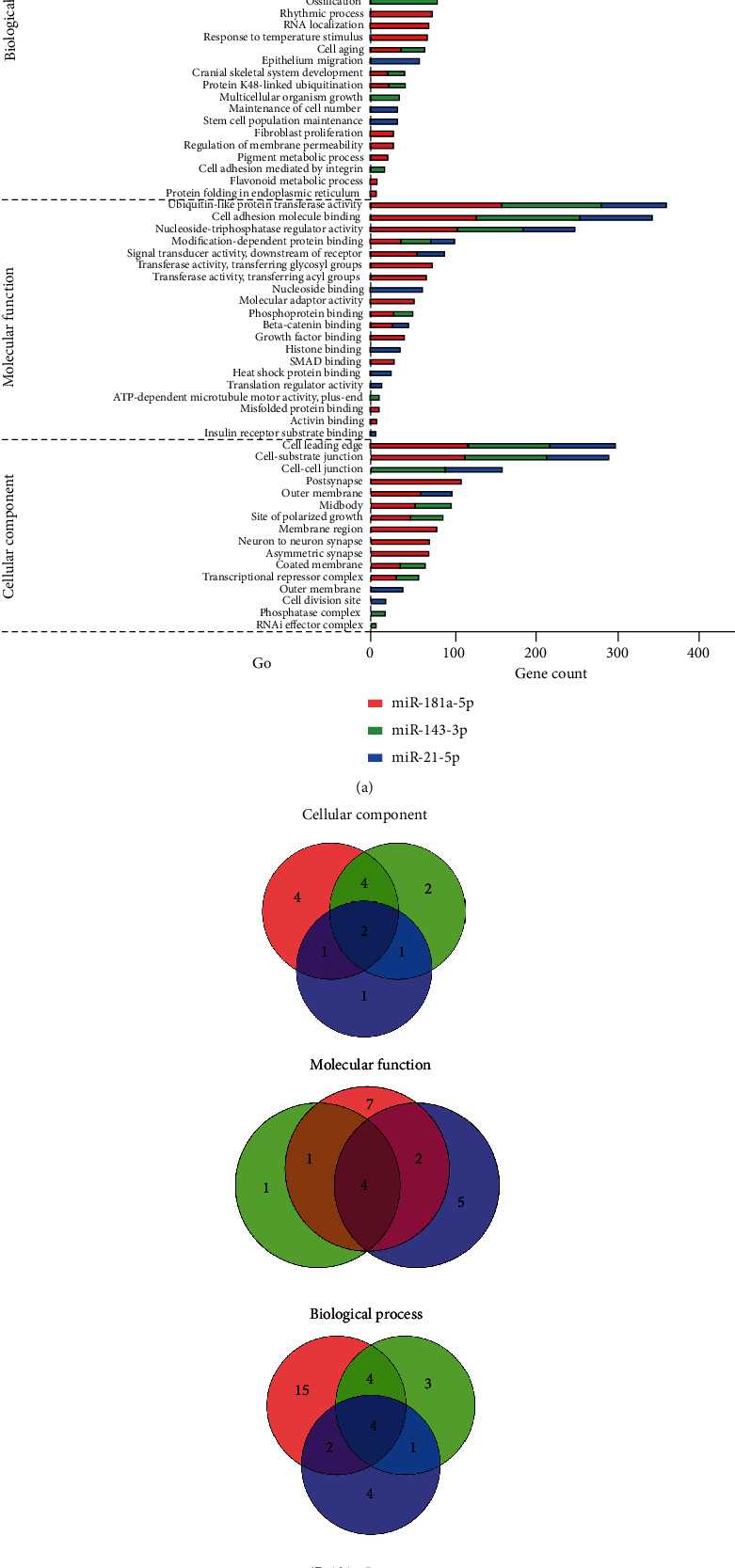
Enriched GO terms of predicted targets of differentially expressed miRNAs. (a) The stacked chart shows significantly enriched GO terms (cellular component, molecular function, and biological process) of predicted targets of miR-181a-5p, miR-143-3p, and miR-21-5p. The gene count indicates the number of target genes that related to each GO term. (b) The Venn graph to show numbers of common GO terms enriched for predicted targets of miR-181a-5p, miR-143-3p, and miR-21-5p. The names of the common GO terms can be checked out in (a).

According to the enriched GO terms in the cellular component, the cell leading edge and cell-substrate junction, of which both are correlated with cell movement and adhesion, were the common enriched terms of all the 3 miRNA target genes (Figure 3). As to molecular functions and biological processes, the 3 miRNA target genes shared common enriched GO terms that were involved in the ubiquitin-like protein transferase activity, nucleoside-triphosphatase regulator activity, cell adhesion, endomembrane system organization, and process utilizing the autophagic mechanism. In brief, the analysis for enriched GO terms of these miRNA target genes indicated that all the 3 miRNAs have common GO terms involved in cell adhesion, endomembrane system activity, and ubiquitylation.

KEGG analysis also showed that there were common pathways among the 3 miRNA target genes (Table 1), for example, endocytosis, cell adhesion, tumor (glioma, renal cell carcinoma, melanoma, prostate cancer, and so on) signaling, and apoptosis. Some molecular pathways, such as the MAPK, ErbB, mTOR, and insulin signaling pathways, were also the common targets of the 3 miRNAs. Among these, the MAPK and ErbB signaling pathways were further confirmed by REACTOME pathway analysis as the common target pathways.

### 3.2. GSEA Showed Dysregulated Membrane Transport, Topological Incorrect Protein Response, Transcription and Translation, and Material Metabolism in Diabetic HDFs

**Figure 4:**
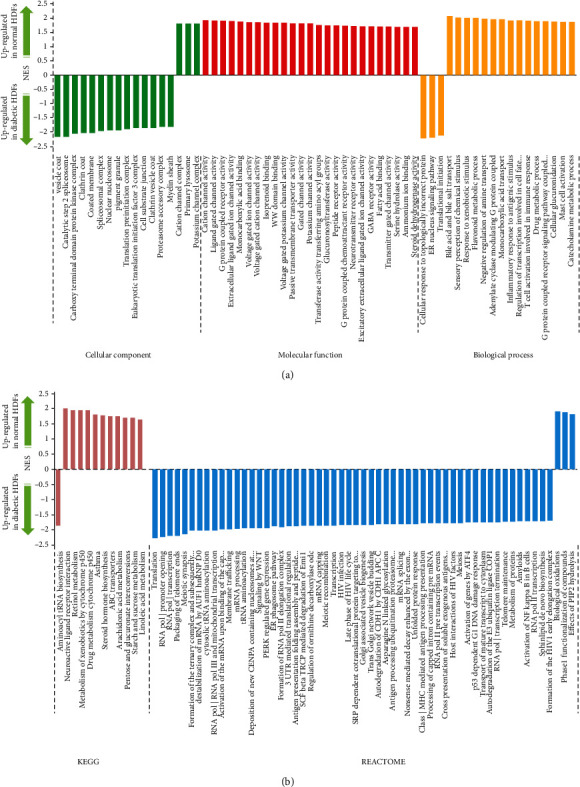
GSEA of the whole mRNA expression profiling data. We use the GO terms, KEGG pathway, and REACTOME pathway as predefined gene sets. Gene sets with FDR < 0.05 were considered significantly enriched. (a) Significantly enriched gene sets relating to GO terms in GSEA. (b) Significantly enriched gene sets relating to the KEGG and REACTOME pathways in GSEA. NES: normalized enrichment score. Generally, the greater absolute value of NES means that the expressions of genes in the gene set are more accordantly changed in experimental conditions. The positive NES value indicates higher expression of gene sets in the normal group. The negative NES value indicates higher expression of gene sets in the diabetic group.

GSEA was carried out using the whole mRNA expression profiling data to further testify the functional changes brought by diabetes in cultured HDFs. We used the GO terms, KEGG pathway, and REACTOME pathway as predefined gene sets (Figure 4). In diabetic HDFs, 14 predefined gene sets relating to GO terms of the cellular component were enriched, including the coated membrane, spliceosomal complex, nuclear nucleosome, and translation preinitiation complex. As to biological process-related GO terms, 3 gene sets of cellular response to topologically incorrect protein, endoplasmic reticulum to nucleus signaling, and translation initiation were enriched. No molecular function-related GO terms were significantly enriched in diabetic HDFs. Based on GSEA of KEGG pathways, we identified that the gene set of aminoacyl-tRNA biosynthesis was significantly enriched in diabetic HDFs. However, REACTOME pathway-based GSEA identified that 54 gene sets that were mainly involved in pathways of gene transcription and translation, membrane trafficking, ubiquitous degradation, unfolded protein response, and antigen presentation, as well as WNT, NF-*κ*B, PERK, and ATF4 signaling, were significantly enriched.

In normal HDFs, 3 GO terms of the cellular component were enriched, including the primary lysosome, potassium, and cation channel complex. 25 GO terms of molecular function were enriched, which were involved in ion channel activity and receptor signaling activity. As to biological process-related GO terms, 14 gene sets were enriched, which were associated with the metabolic process, response to chemical and antigenic stimuli, and G protein-coupled receptor signaling pathways. GSEA of KEGG pathways identified that 11 gene sets, involving metabolism (of nutrients, steroids, retinol, and drugs), ABC transporters, neuroactive ligand-receptor interaction, and asthma, were significantly enriched in normal HDFs. The 3 REACTOME gene sets significantly enriched were pathways of biological oxidations, phase 1 functionalization of compounds (which is closely related to material metabolism), and effects of PIP2 hydrolysis.

Collectively, the functions of genes enriched in diabetic HDFs were mainly attributed to membrane transport, topologically incorrect protein, transcription, and translation. Nevertheless, the functions of genes enriched in normal HDFs were mainly attributed to material metabolism, ion channel activity, and G protein-coupled receptor signaling pathways.

### 3.3. Gene Correlation Network Analysis Suggested That miR-181a-5p Plays a Key Role among the DEGs

**Figure 5:**
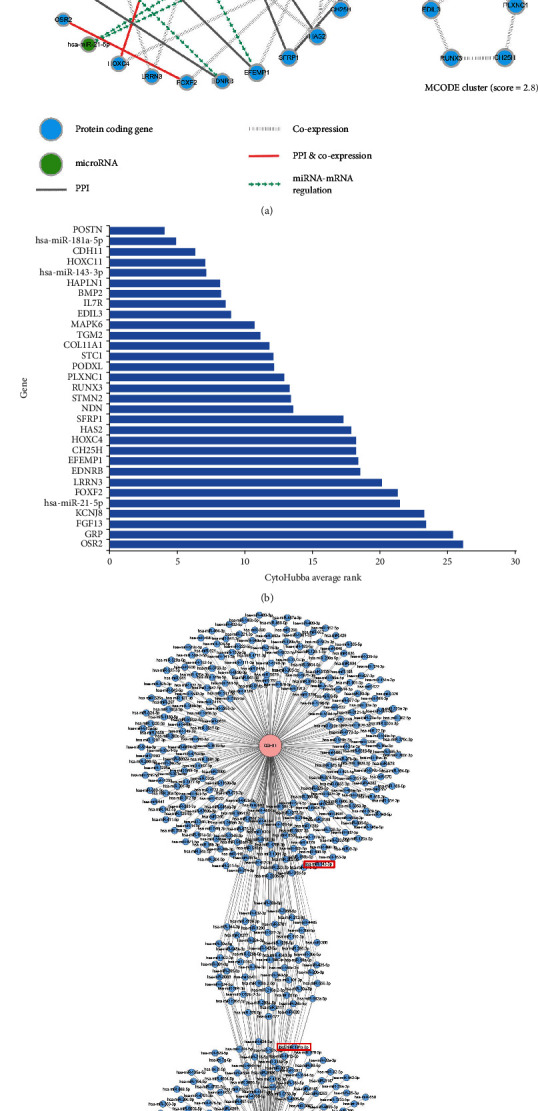
Gene correlation network analysis to identify hub genes of HDFs in the diabetic skin. (a) The relationships among the differentially expressed mRNAs and miRNAs. The relationship of protein-protein interactions (PPI), coexpression, coexistence of PPI and coexpression, and predicated miRNA-mRNA regulation is presented by different line types. The MCODE cluster suggested a close relationship of POSTN and CDH11 in a network cluster view. (b) The CytoHubba average rank of differentially expressed miRNAs and mRNAs. The smaller the average rank is, the higher the importance of the gene is. (c) The miRNA network that may affect the expressions of hub protein-coding genes (POSTN, CDH11). The cluster in the middle of graph (c) shows the miRNAs that control the expression of both genes. The red boxes indicate the differentially expressed miRNAs of miR-181a-5p and miR-143-3p.

To visualize the whole relationship of DEGs, the gene correlation network of DEGs was constructed by merging the PPI network, weighted gene coexpression network, and miRNA-mRNA target regulatory network. As shown in Figure 5(a), the network consists of 30 genes and 68 gene-gene correlations. In the gene correlation network, miR-181a-5p had the largest node degree, which suggested that miR-181a-5p correlates with the largest number of DEGs. By combining the average rank method and the 12 different built-in scoring methods, CytoHubba identified POSTN, miR-181a-5p, and CDH11 as the top 3 hub genes (Figure 5(b)). Furthermore, through analysis using the MCODE program, we identified a cluster of genes that were the most correlated with each other, including POSTN, CDH11, EDIL3, PLXNC1, CH25H, and RUNX3. This result suggested that the hub gene of POSTN and CDH11 may work as a functional group in HDFs. As shown in Figures 5(a) and 5(c), miR-181a-5p was predicted to control the expression of POSTN. Therefore, we could infer from the gene correlation network analysis that as a miRNA which modulates the POSTN/CDH11 function, miR-181a-5p possibly plays a key role in modulation of HDF behavior during the diabetic state.

### 3.4. Validation of Differential Expression of miRNAs and mRNAs in High Glucose-Cultured HDFs

**Figure 6:**
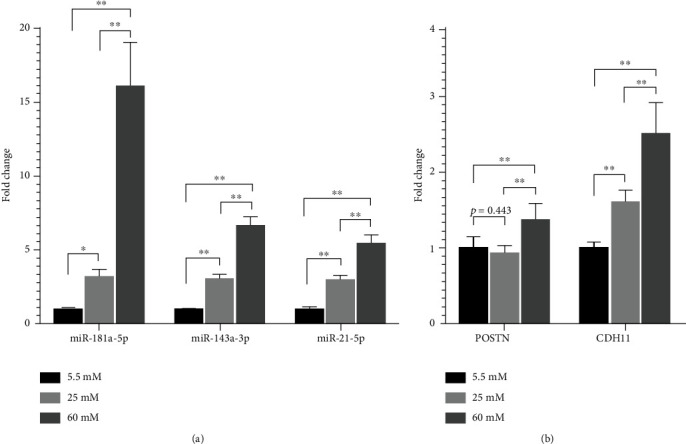
Validation of differential expression of several miRNAs and mRNAs in different glucose concentration-cultured HDFs. (a) Expression of miR-181a-5p, miR-143a-5p, and miR-21-5p in different glucose concentration-cultured HDFs (*n* = 6). (b) Expression of POSTN and CDH11 mRNAs in different glucose concentration-cultured HDFs (*n* = 6). The fold changes were calculated by comparing to the normal glucose (5.5 mM) group. ^∗^*p* < 0.05, ^∗∗^*p* < 0.01.

To verify the expression patterns of differentially expressed genes, we performed qRT-PCR analysis to determine the levels of differentially expressed miRNAs (miR-181a-5p, miR-143-3p, and miR-21-5p) and mRNAs levels of hub protein-coding genes (POSTN, CDH11) in different concentrations of glucose-cultured HDFs Figure 6. Compared with normal glucose (5.5 mM), high-glucose treatment in HDFs significantly upregulated the level of miR-181a-5p (increased to 3.18 folds in 25 mM of glucose, *p* = 0.04, and to 16.10 folds in 60 mM of glucose, *p* < 0.01), miR-143-3p (increased to 3.04 folds in 25 mM of glucose, *p* < 0.01, and to 6.66 folds in 60 mM of glucose, *p* < 0.01), and miR-21-5p (increased to 2.96 folds in 25 mM of glucose, *p* < 0.01, and to 5.41 folds in 60 mM of glucose, *p* < 0.01). Although in the high-glucose level of 25 mM, no significant influence on POSTN mRNA expression has been reached, 60 mM of glucose treatment could significantly increase the POSTN mRNA level to 1.36 folds (*p* < 0.01). Furthermore, both 25 mM and 60 mM of glucose treatment significantly upregulated the CDH11 mRNA level to 1.61 folds (*p* < 0.01) and 2.50 folds (*p* < 0.01), when compared to normal glucose treatment. All the expression trends of differentially expressed miRNAs and hub genes in high glucose-cultured HDFs were consistent with our differential expression analysis results. Thus, in vitro qRT-PCR experiments verified the differential expression analyses.

## 4. Discussion

As a major cell that maintains connective tissue homeostasis, dermal fibroblasts are deeply involved in the wound healing process. They migrate and proliferate to form granulation tissue, differentiate into myofibroblasts for wound contraction, and produce and degrade the extracellular matrix for wound remodeling. The impaired dermal fibroblast function during diabetic wound healing has long been recognized. However, few studies have focused on the discovery and interpretation of genetic basis that underlies the dermal fibroblast dysfunction. Therefore, based on multiple bioinformatics analysis methods and in vitro experiments, the present study was carried out to further dig and interpret the published miRNA and mRNA profiling data.

According to our analysis of profiling data, in diabetic HDFs, translation of genes such as POSTN, CDH11, EDIL3, IL7R, COL11A1, HAPLN1, and MAPK6 is upregulated, and genes like BMP2, STMN2, and SFRP1 are downregulated. The encoded proteins of most these genes participate in the formation of the extracellular matrix, cell migration, proliferation, and angiogenesis, which are required for normal wound healing. For example, POSTN and CDH11, which encode cell adhesion molecules and induce cell attachment and spreading, are required for cell proliferation, migration, differentiation, and ECM remodeling. COL11A1 and HAPLN1, respectively, encode collagen type XI alpha 1 chain and hyaluronan and proteoglycan link protein 1, which are components of ECM and play important roles in tissue development. Moreover, the downregulated genes of STMN2 and SFRP1 encode proteins that can affect cell growth and angiogenesis during ischemic injury.

We also found 3 differentially expressed miRNAs, miR-181a-5p, miR-143-3p, and miR-21-5p. Although few studies have focused on the expression and functions of the 3 miRNAs in diabetic HDFs, they all have been reported as relevant to diabetes. In regard to miR-181a-5p, it has been found to have antiproliferation, antifibrotic, and anti-inflammatory effects in many cell types [11–14], as well as in the diabetic state [11, 12]. According to 2 independent studies carried out by Liu et al. [15] and Wei et al. [16], miR-181a-5p has the ability to reduce endoplasmic reticulum (ER) stress through glucose-regulated protein, 78 kDa (GRP78), which is a major ER chaperone and signaling regulator and has been identified as a target of miR-181a-5p. As to miR-143-3p, the correlations of its overexpression and diabetes have been proved by Massaro et al. [17] and Dahlman et al. [18]. In the light of previous studies, miR-143-3p can inhibit tumor cell proliferation, migration, and invasion in many different malignant tumors, such as melanoma [19], osteosarcoma [20], and lung cancer [21]. The question whether miR-143-3p works in a similar way in diabetic HDFs still needs further investigation. As to miR-21-5p, a few studies have reported that its overexpression was associated with diabetic complications, especially diabetic nephropathy. The antiangiogenesis and antiproliferation effects of miR-21-5p are probably responsible for its involvement in diabetic complications. Therefore, we found that the 3 miRNAs have overlapped function of repressing cell proliferation, which is an important process during wound healing. Considering the above mentioned functions of differentially expressed genes and in vitro validation results, it is reasonable to infer that the proliferative ability of HDF is impaired in high-glucose condition during diabetes.

Seeing that we have applied all the DEG enrichment analysis, miRNA target enrichment analysis, and GSEA, we attempted to summarize the results of the 3 analyses. Collectively, when all the enrichment results are taken into consideration, the enriched pathways are closely correlated with enriched GO terms. Similar terms of enriched pathways, for example, extracellular matrix organization, endocytosis, cell adhesion, metabolism, transcription, and translation, also occurred in GO enrichment analyses. However, the enriched GO terms of the 3 analyses are literally different from each other. Among the involved aspects of GO terms from all 3 analyses, formation of ECM, cell adhesion, proliferation, differentiation, and hypoxia response can be seen as consequences of membrane transport, topological incorrect protein response (including ubiquitylation and endocytosis), material metabolism, transcription, and translation. This suggests to us that the alterations to formation of ECM, cell adhesion, proliferation, differentiation, and hypoxia response are notable functional changes in diabetic HDFs. Deregulations of membrane transport, topological incorrect protein response, material metabolism, transcription, and translation are basic mechanisms that underlie these changes. Indeed, diabetic dermal fibroblasts had shown decreased collagen synthesis [22] and increased MMP production [23] compared with normal dermal fibroblasts. As the synthesis and secretion process of these proteins requires transcription, translation, membrane transport, and material metabolism, there is no doubt that they are dysregulated in diabetic HDFs. Response to topological incorrect protein involves ubiquitylation and ER stress [24]. Moreover, ER stress can decrease protein synthesis, promote protein degradation, and further induce autophagy [25] in dermal fibroblasts. A recent study [15] has reported that miR-181a-5p reduces ER stress, which may address the key role of miR-181a-5p in maintaining material and energy homeostasis in diabetic HDFs.

Our study has identified 3 hub genes including POSTN, miR-181a-5p, and CDH11. Periostin, the coding protein of POSTN, has already been recognized as an important regulator during diabetic wound healing [26]. It induces the proliferation of fibroblasts and is involved in the TGF-*β*-provoked induction of myofibroblasts during wound healing. The CDH11 coding protein, cadherin 11, is also reported to be a regulator of wound healing and tissue regeneration [27]. Our in vitro experiment verified the overexpression of POSTN and CDH11 in high glucose (60 mM)-cultured HDFs. Therefore, it is not surprising that POSTN and CDH11 are hub genes in the regulation of diabetic HDFs. According to our analysis, POSTN is a predicted target of miR-181-5p. Correspondingly, in vitro experiment further confirmed the overexpression of miR-181a-5p, as well as a slight downregulation of POSTN, in high glucose (25 mM)-cultured HDFs. Higher glucose (60 mM) increased both miR-181a-5p and POSTN expression, which is probably due to a comprehensive regulation of POSTN by other regulators. Previous studies have reported the elevation of miR-181a-5p in serum of diabetic patients [28] and in separated HUVECs of diabetic rats [29]. Moreover, inhibition of endogenous miR-181a-5p could improve insulin sensitivity [28]. Thus, miR-181a-5p is closely related to diabetes. Considering that miR-181a-5p also participates in the modulation of cell migration [13], fibrosis [12], and ER stress [24], which are deeply involved in the wound healing process, it is reasonable to speculate miR-181a-5p as a key regulator in diabetic HDFs.

The limit of the present study is that we did not make further experiments to explore how these hub genes participate in the regulation network of diabetic HDFs. Our future work will focus on exploring the effects and mechanisms of miR-181a-5p, POSTN, and CDH11 in high glucose-cultured HDFs.

## 5. Conclusions

In the present study, we identified that 3 miRNAs and 30 mRNAs were differentially expressed in diabetic skin-derived HDFs. These genes are involved in ECM organization, angiogenesis, cell proliferation, and migration, which are closely related to wound healing. Enrichment analyses showed deregulation of ECM organization, cell adhesion, proliferation, differentiation, hypoxia response, membrane transport, topological incorrect protein response, material metabolism, transcription, and translation. Based on the gene correlation network, we identified the top 3 hub genes: miR-181a-5p, POSTN, and CDH11. Among these, POSTN is a predicted target of miR-181a-5p and is supposed to work together with CDH11 as a functional group. Therefore, miR-181a-5p possibly plays a key role in modulation of HDF behavior during the diabetic state. Although the expression pattern of the hub genes was verified by in vitro quantification experiments in high glucose-cultured HDFs, more experimental studies are needed to further confirm our present findings. Our future work will focus on exploring the effects and mechanisms of miR-181a-5p in high glucose-cultured HDFs, which can lay the theoretical and experimental base for the targeted therapeutic strategy to treat the diabetic wound.

## Figures and Tables

**Table 1:** Common pathways among target genes of the 3 differentially expressed miRNAs.

Category	ID	Description
KEGG	hsa04144	Endocytosis
	hsa05200	Pathways in cancer
	hsa04910	Insulin signaling pathway
	hsa05214	Glioma
	hsa05211	Renal cell carcinoma
	hsa05215	Prostate cancer
	hsa04520	Adherens junction
	hsa04141	Protein processing in the endoplasmic reticulum
	hsa04012	ErbB signaling pathway
	hsa04722	Neurotrophin signaling pathway
	hsa04010	MAPK signaling pathway
	hsa04150	mTOR signaling pathway
	hsa04810	Regulation of actin cytoskeleton
	hsa05220	Chronic myeloid leukemia
	hsa05223	Non-small-cell lung cancer
	hsa04510	Focal adhesion
	hsa04070	Phosphatidylinositol signaling system
	hsa03018	RNA degradation
	hsa05212	Pancreatic cancer
	hsa00562	Inositol phosphate metabolism
	hsa05210	Colorectal cancer
	hsa05100	Bacterial invasion of epithelial cells
	hsa04210	Apoptosis
	hsa05142	Chagas disease (American trypanosomiasis)
	hsa05131	Shigellosis
REACTOME	R-HSA-5663202	Diseases of signal transduction
	R-HSA-9006934	Signaling by receptor tyrosine kinases
	R-HSA-6802957	Oncogenic MAPK signaling
	R-HSA-9006936	Signaling by TGF-*β* family members
	R-HSA-187037	Signaling by NTRK1 (TRKA)
	R-HSA-450294	MAP kinase activation
	R-HSA-1257604	PIP3 activates AKT signaling
	R-HSA-166520	Signaling by NTRKs
	R-HSA-1236394	Signaling by ERBB4
	R-HSA-168138	Toll-like receptor 9 (TLR9) cascade

## Data Availability

The raw data used in our study are freely available from GEO datasets GSE68185, GSE84971, GSE49566, and GSE78891.

## References

[1] Trautner C., Haastert B., Mauckner P., Gatcke L. M., Giani G. (2007). Reduced incidence of lower-limb amputations in the diabetic population of a German city, 1990-2005: results of the Leverkusen Amputation Reduction Study (LARS). *Diabetes Care*.

[2] Khanolkar M. P., Bain S. C., Stephens J. W. (2008). The diabetic foot. *QJM*.

[3] Jhamb S., Vangaveti V. N., Malabu U. H. (2016). Genetic and molecular basis of diabetic foot ulcers: clinical review. *Journal of Tissue Viability*.

[4] Millioni R., Puricelli L., Iori E., Trevisan R., Tessari P. (2012). Skin fibroblasts as a tool for identifying the risk of nephropathy in the type 1 diabetic population. *Diabetes/Metabolism Research and Reviews*.

[5] Caramori M. L., Kim Y., Moore J. H. (2012). Gene expression differences in skin fibroblasts in identical twins discordant for type 1 diabetes. *Diabetes*.

[6] Li J. H., Liu S., Zhou H., Qu L. H., Yang J. H. (2014). starBase v2.0: decoding miRNA-ceRNA, miRNA-ncRNA and protein-RNA interaction networks from large-scale CLIP-Seq data. *Nucleic Acids Research*.

[7] Hu W., Ding Y., Wang S., Xu L., Yu H. (2020). The construction and analysis of the aberrant lncRNA-miRNA-mRNA network in adipose tissue from type 2 diabetes individuals with obesity. *Journal of Diabetes Research*.

[8] Nasr M. B., Tezza S., D’Addio F. (2017). PD-L1 genetic overexpression or pharmacological restoration in hematopoietic stem and progenitor cells reverses autoimmune diabetes. *Science Translational Medicine*.

[9] Paraskevopoulou M. D., Georgakilas G., Kostoulas N. (2013). DIANA-microT web server v5.0: service integration into miRNA functional analysis workflows. *Nucleic Acids Research*.

[10] Sticht C., De La Torre C., Parveen A., Gretz N. (2018). miRWalk: an online resource for prediction of microRNA binding sites. *PLoS One*.

[11] Hu W., Yan F., Ru Y. (2020). MIIP inhibits EMT and cell invasion in prostate cancer through miR-181a/b-5p-KLF17 axis. *American Journal of Cancer Research*.

[12] Xu P., Guan M. P., Bi J. G., Wang D., Zheng Z. J., Xue Y. M. (2017). High glucose down-regulates microRNA-181a-5p to increase pro-fibrotic gene expression by targeting early growth response factor 1 in HK-2 cells. *Cell Signal*.

[13] Wu L., Song W. Y., Xie Y. (2018). miR-181a-5p suppresses invasion and migration of HTR-8/SVneo cells by directly targeting IGF2BP2. *Cell Death & Disease*.

[14] Su Y., Yuan J., Zhang F. (2019). MicroRNA-181a-5p and microRNA-181a-3p cooperatively restrict vascular inflammation and atherosclerosis. *Cell Death & Disease*.

[15] Liu J., Huang Y., Cai F., Dang Y., Liu C., Wang J. (2020). MicroRNA-181a regulates endoplasmic reticulum stress in offspring of mice following prenatal microcystin-LR exposure. *Chemosphere*.

[16] Wei Y., Tao X., Xu H. (2016). Role of miR-181a-5p and endoplasmic reticulum stress in the regulation of myogenic differentiation,. *Gene*.

[17] Massaro J. D., Polli C. D., e Silva M. C. (2019). Post-transcriptional markers associated with clinical complications in type 1 and type 2 diabetes mellitus. *Molecular and Cellular Endocrinology*.

[18] Dahlman I., Belarbi Y., Laurencikiene J., Pettersson A. M., Arner P., Kulyte A. (2017). Comprehensive functional screening of miRNAs involved in fat cell insulin sensitivity among women. *American Journal of Physiology-Endocrinology and Metabolism*.

[19] Panza E., Ercolano G., De Cicco P. (2018). MicroRNA-143-3p inhibits growth and invasiveness of melanoma cells by targeting cyclooxygenase-2 and inversely correlates with malignant melanoma progression. *Biochemical Pharmacology*.

[20] Hou Y., Feng H., Jiao J. (2019). Mechanism of miR-143-3p inhibiting proliferation, migration and invasion of osteosarcoma cells by targeting MAPK7. *Artificial Cells, Nanomedicine, and Biotechnology*.

[21] Wang H., Deng Q., Lv Z. (2019). N6-methyladenosine induced miR-143-3p promotes the brain metastasis of lung cancer via regulation of VASH1. *Molecular Cancer*.

[22] Xu J., Zgheib C., Hodges M. M., Caskey R. C., Hu J., Liechty K. W. (2017). Mesenchymal stem cells correct impaired diabetic wound healing by decreasing ECM proteolysis. *Physiol Genomics*.

[23] Wall S. J., Sampson M. J., Levell N., Murphy G. (2003). Elevated matrix metalloproteinase-2 and -3 production from human diabetic dermal fibroblasts. *British Journal of Dermatology*.

[24] Ishikawa T., Toyama T., Nakamura Y. (2017). UPR transducer BBF2H7 allows export of type II collagen in a cargo- and developmental stage-specific manner. *Journal of Cell Biology*.

[25] Umar S. A., Tanveer M. A., Nazir L. A., Divya G., Vishwakarma R. A., Tasduq S. A. (2019). Glycyrrhizic acid prevents oxidative stress mediated DNA damage response through modulation of autophagy in ultraviolet-B-irradiated human primary dermal fibroblasts. *Cellular Physiology and Biochemistry*.

[26] Murota H., Lingli Y., Katayama I. (2017). Periostin in the pathogenesis of skin diseases. *Cellular and Molecular Life Sciences*.

[27] Row S., Liu Y., Alimperti S., Agarwal S. K., Andreadis S. T. (2016). Cadherin-11 is a novel regulator of extracellular matrix synthesis and tissue mechanics. *Journal of Cell Science*.

[28] Zhou B., Li C., Qi W. (2012). Downregulation of miR-181a upregulates sirtuin-1 (SIRT1) and improves hepatic insulin sensitivity. *Diabetologia*.

[29] Zhang Q., Xiao X., Zheng J., Li M. (2019). A glucagon-like peptide-1 analog, liraglutide, ameliorates endothelial dysfunction through miRNAs to inhibit apoptosis in rats. *PeerJ*.

